# Molecular detection of Citrus exocortis viroid (CEVd), Citrus viroid-III (CVd-III), and Citrus viroid-IV (CVd-IV) in Palestine

**DOI:** 10.1038/s41598-023-50271-5

**Published:** 2024-01-03

**Authors:** Aswar Abualrob, Osama Alabdallah, Raied Abou Kubaa, Sabri M. Naser, Raed Alkowni

**Affiliations:** 1 Biology and Biotechnology Department, An-Najah National University, Nablus, Palestine; 2 National Agricultural Research Center (NARC), Jenin, Palestine; 3 Department of Plant Pathology, University of California, Davis, CA 95616 USA

**Keywords:** RNA sequencing, Biotechnology

## Abstract

Citrus hosts various phytopathogens that have impacted productivity, including viroids. Missing data on the status of viroids in citrus in Palestine were not reported. This study was aimed to detect any of *Citrus exocortis viroid* (CEVd), *Citrus viroid-III* (CVd-III), and *Citrus viroid-IV* (CVd-IV) in the Palestinian National Agricultural Research Center (NARC) germplasm collection Field inspections found symptoms such as leaf epinasty; vein discoloration, and bark cracking on various citrus varieties. RT-PCR revealed a significant prevalence of CVd-IV; CEVd and CVd-III (47%, 31%, and 22%; respectively). CVd-III variants with 91.3% nucleic acid sequence homology have been reported. The sequence of each viroid were deposited in GenBank as (OP925746 for CEVd, OP902248 and OP902249 for CVd-III-PS-1 and -PS-2 isolates, and OP902247 for CVd-IV). This was the first to report three of citrus viroids in Palestine, appealing to apply of phytosanitary measures to disseminate healthy propagating materials free from viroids.

## Introduction

Citrus is a host to several pathogens, many of which have an economic impact on the crop. Viroids are among of these induced annual worldwide losses in fruit trees^1,2^. They are known as causal agents of many diseases on herbaceous and woody plants^3^ causing symptoms on leaves (deformation, vein discoloration, epinasty, mottling, chlorotic and necrosis), stems (cankers, bark scaling and/or cracking), fruits malformation, and in severe cases plants death^4^.

The *Potato spindle tuber viroid* was the first detected on potato plants in 1971 by Danier^5^, before many viroids were reported infecting several plant crops worldwide, particularly in warm climate regions^6^.

To date, eight viroid species belonging to the Pospiviroidae family have been reported in citrus: *Citrus exocortis viroid* (CEVd), *Hop stunt viroid* (HSVd), *Citrus viroid-III* (CVd-III), *Citrus viroid-IV* (CVd-IV; syn. *Citrus bark cracking viroid* (CBCVd), *Citrus viroid-V* (CVdV), *Citrus viroid-VI* (CVd-VI), *Citrus viroid-VII* (CVd-VII), *Citrus dwarfing viroid* (CDVd), and *Citrus bent leaf viroid* (CBLVd)^7–12^.

The main constraints on the use of PCR in diagnosis are the cost and availability of sufficient sample materials which multiplex PCR can overcome^13^. Multiplex PCR enables the amplification of multiple target sequences by employing more than one pair of primers in the reaction. Multiplex PCR, with no loss of test utility, has the potential to result in significant time and labor savings in the laboratory. It has been successfully used in a variety of nucleic acid diagnostic applications, including RNA detection, quantitative analysis, mutation and polymorphism analysis, gene deletion analysis, and many others^14^. The technique has been demonstrated to be a valuable method for identifying viruses, bacteria, fungi, and other microbes in the field of infectious diseases. CVd-IV (*syn*. CBCVd) was considered as minor pathogen in the genus *Citrus*^11^, but it was associated with severe bark cracking on trifoliate orange rootstock^15–17^. CEVd was known to have a wide host range^6,18,19^, and symptoms in different species of citrus, such as stunting, bark sloughing and cracking, leaf epinasty and cracks in the petiole^20–25^. CVd-III was reported to have of particular interest due to its genomic variability beside its significant reduction of growth and yield^26–29^.

Since no data on the existence of citrus viroids in the Palestinian territories was available, research on their existence and prevalence was initiated as part of a collaboration with the National Agricultural Research Center for eight viroids. The purpose of this study was to determine the presence and prevalence of three viroids (CEVd; CVd-III; and CVd-IV) in Palestinian germplasm collection and to be the first report in this region.

## Results and discussion

### Recognition of putatively viroid’s symptoms

**Figure 1:**
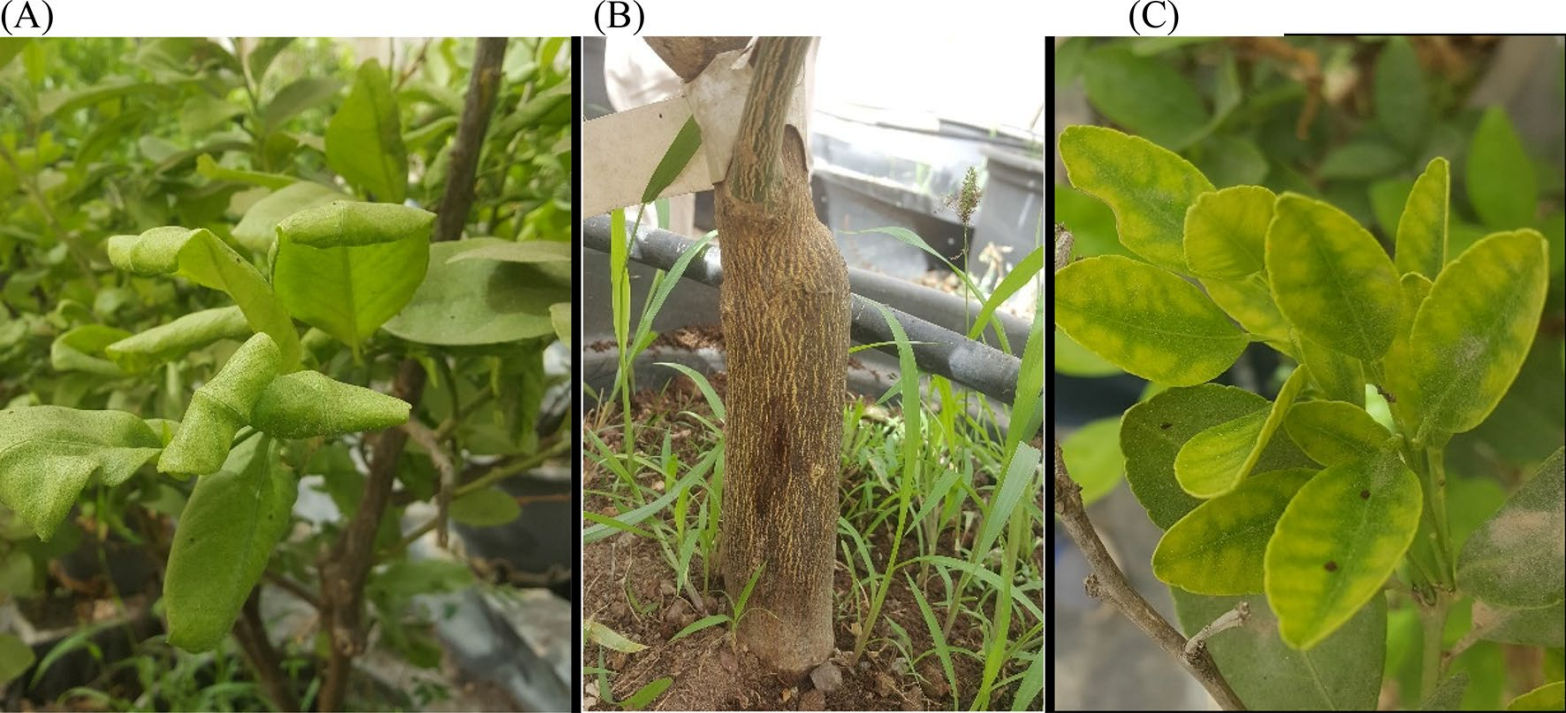
Inspected citrus trees were exhibited symptoms as leaf epinasty on Pomelo (**A**); cankers of bark on Clementine (**B**); and leaf discoloration on Lemon (**C**), of putatively viroid’s pathogeneses.

No data was available about the existence of citrus viroids in the country. However, many reported in the region^6,30–33^. Lacking specific symptoms; which are sometimes symptomless or similar to those of virus sources, it could be hard to specifically determine the viroid-infected trees^34^. Field inspections were carried out on citrus germplasm collection at the National Agriculture Research Center (NARC), Jenin-Palestine. Putative viroid like symptoms such as epinasty, stunting, vein clearing, discoloration, distortion of leaves, mottling, necrotic or chlorotic spots, scaling, cankers, and bark cracking were reported (Fig. 1). These observations nudge the believe the existence of viroid infections in the germplasm collection which might be virus free/tested ones.

### RT-PCR detection

**Figure 2:**
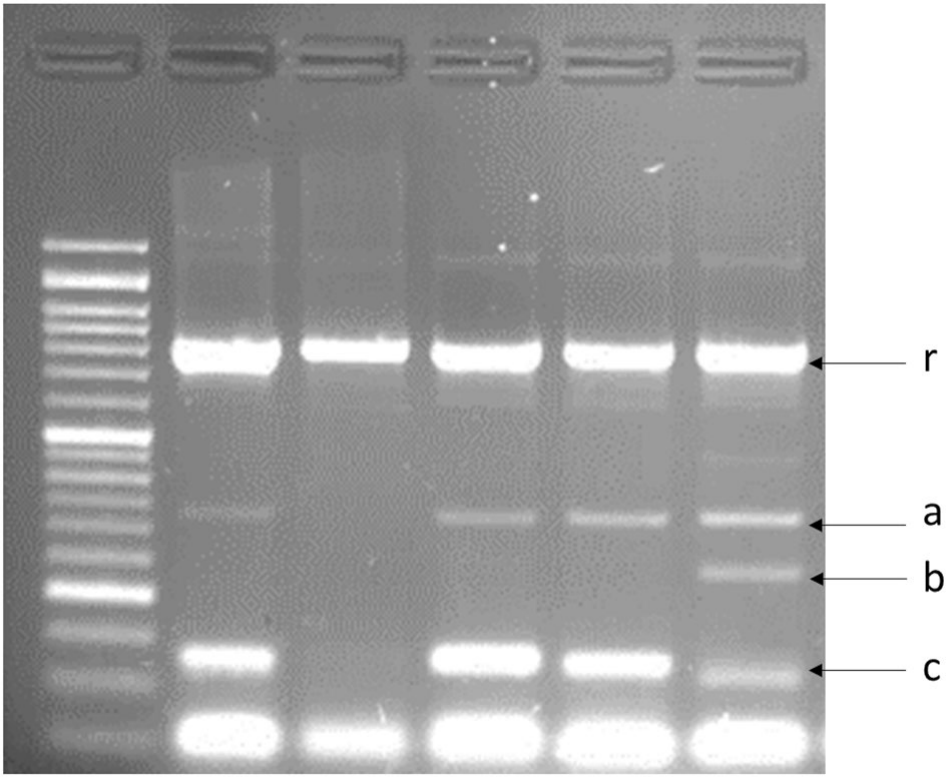
Specific sets of primers were able to amplify the expected portion of the citrus viroid species: CEVd (**a**); CVd-III (**b**); and CVd-IV (**c**) from infected citrus plant samples by single RT-PCR, as well as multiplex one. Plant rRNA primers (r) were applied to amplify portion of 18s rRNA as an internal positive control. 50 bp DNA Ladder RTU (Bio-Helix) was used as a molecular marker.

All these selected three viroids were ensured through molecular detection in a single RT-PCRs as well as multiplex PCR, which successfully applied to all tested viroids. multiplex PCR was performed successfully, and thus it could be recommended as quick and efficient tool for simultaneous detection of viroids at one reaction (Fig. 2).

**Figure 3:**
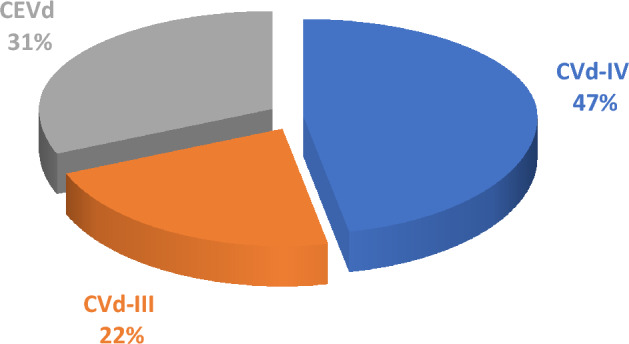
Prevalence of viroids in the citrus as detected by RT-PCR.

Surprisingly, a relatively high total prevalence of infection of these three citrus viroids was reported (52.4%), of which CVd-IV was having the highest incidence (Fig. 3), even though it was found to be less frequent in mixed infections (Table 1).

**Table 1:** Number of detected samples with CVd-IV, CEVd and CVd-III in germplasm collection plot.

Citrus cultivar	Number of tested samples	Number of healthy samples	Number of infected samples					
			*CVd-IV*	*CEVd*	*CVd-III*	Single	Double	Treble
Clementine	16	8	6	2	2	7	0	1
Orange	11	8	1	2	1	2	1	0
Pomelo	4	2	2	1	0	1	1	0
Grapefruit	3	0	2	1	1	2	1	0
Lemon	3	1	1	2	2	0	1	1
Kumquats	3	1	1	2	1	0	2	0
Volkameriana	1	0	1	0	0	1	0	0
Trifoliata	1	0	1	0	0	1	0	0
Total	42	20	15	10	7	14	6	2

Interestingly, samples from citrus rootstocks (Volkameriana and Trifoliata) were found infected with CVd-IV. This might explain high prevalence of the viroid among citrus in Palestine, as well as it has a wide host range^35^. CVd-IV was reported to be one of the main viroids circulating in all citrus-growing areas worldwide^34,36^.

CEVd, one of the most well-known viroid in citrus, was detected in 23.8% of tested samples (Fig. 3). This result was expected since it was found in many countries in Africa (South Africa, Ghana, Sudan, Morocco, Algeria, Tunisia, Libya and Egypt) and in Middle East (Turkey, Cyprus, Israel, Saudi Arabia and Oman)^30,31,33,37–39^.

These viroids were found in single and mixed infections. However, mixed infections could be the most likely cause, which magnifies the severity of the symptoms and reduce the productivity of citrus trees^36^.

### Sequence analysis

The obtained sequences from the viroids (CVd-IV, CEVd, and CVd-III) were BLASTn searched to reveal high similarity with each corresponding viroids in the GenBank data. The sequences were deposited at GenBank under the accession numbers: OP902247 (CVd-IV), OP925746 (CEVd), OP902248 (CVd-III-PS-isolate-1) and OP902249 (CVd-III-PS-isolate-2).

**Figure 4:**
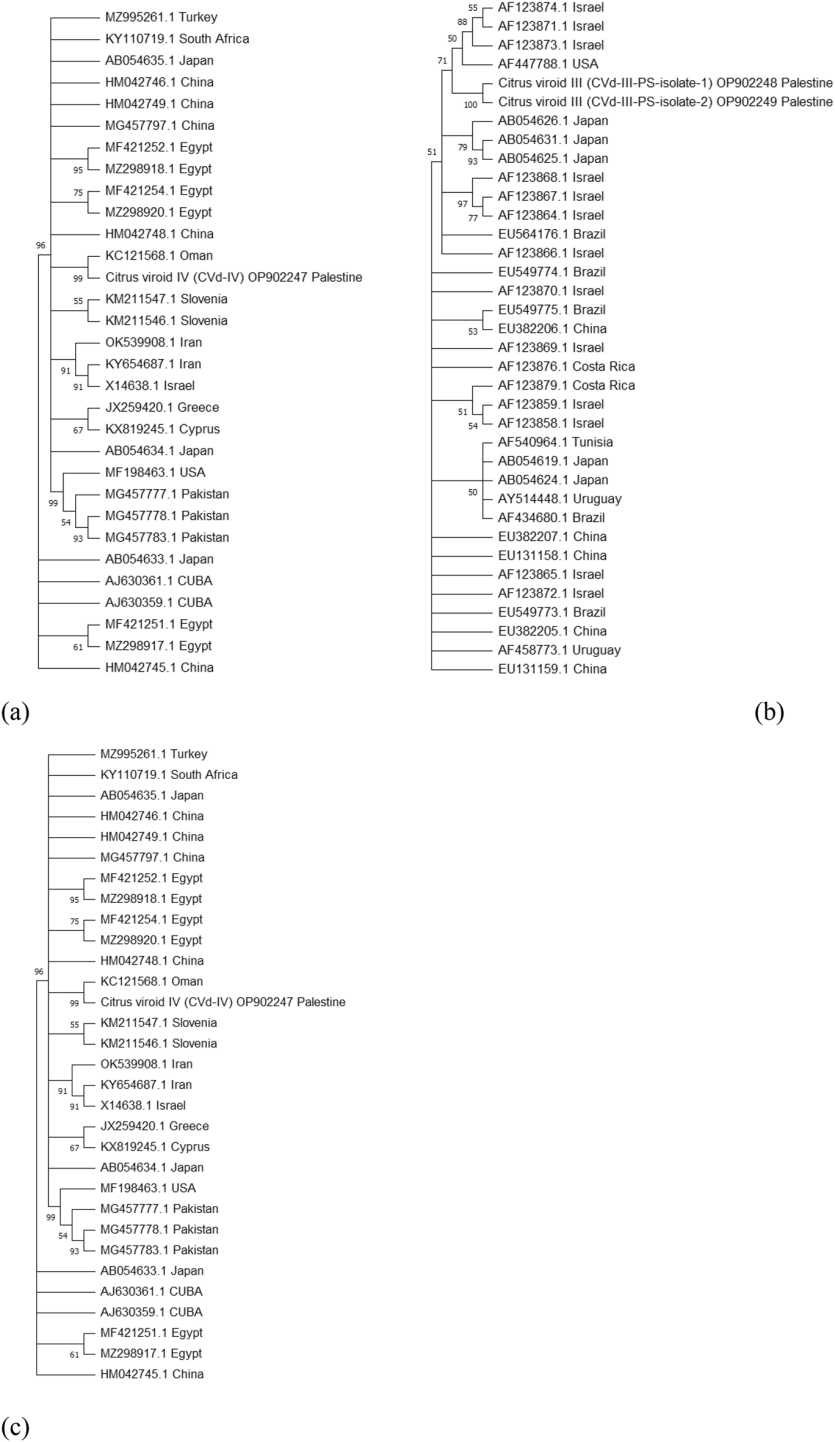
Phylogenetic analysis of (**a**) Citrus viroid IV (CVd-IV) [Accession No.: OP902247]; (**b**) Citrus viroid III (CVd-III) [Accession No.: OP902248 and OP902249] and (**c**) Citrus exocortis viroid (CEVd) [Accession No.: OP925746] sequences against corresponding viroids accessions available in GenBank. The phylogenetic tree was generated by MEGA-11 program with evolutionary history inferred by the Neighbor-Joining in the bootstrap clustering test of 1,000 replicates. The percentage of replicated trees less than 50% was collapsed.

Even though BLASTn searching of the CVd-IV revealed high similarity to those from Turkey (MZ995261) and Oman (KC121568), in accordance with what was proposed for their middle East origin^31^. A phylogenetic tree, constructed using molecular evolutionary genetics analysis across computing platforms (MEGA-11)^40^, revealed that CVd-IV clustered with other similar viroids available in GenBank records (Fig. 4a).

Nucleotide sequences obtained from CVd-III infected samples, two isolates were revealed to have 91.3 percent similarity by Pairwise Sequence Alignment-EMBOSS. Water provided by the European Molecular Biology Laboratory (EMBL); suggesting they were putative locally mutated. Thus, these two isolate considered varied within the viroid genetic variation in the country and designated as CVd-III-PS-isolate-1 and CVd-III-PS-isolate-2. They were clustered together in a phylogenetic tree with other GenBank deposited isolates from the Middle East region, as well as others isolated worldwide (Fig. 4b). The same can be noticed for the CEVd as it aligns with those isolate accessions retrieved from the GenBank (Fig. 4b,c).

Although, this study was the first to report citrus viroid in Palestine, their existence in relatively high prevalence is alarming, especially once rootstocks were detected infected. Knowing their main route of dissemination throughout propagating materials^21,41^; phytosanitary actions are highly recommended, which is the only way to prevent further spread of viroids^42,43^. Certification program and sanitary actions to exclude these viroids side by side with viruses are highly appealing. National germplasm collections must be subjected to strictly measurement to ensure distributing propagating plant materials free of viruses as well as virus like pathogens.

## Methods

### Field inspections

Several field visits to the germplasm collection of the National Agricultural Research Center were achieved since it was established in 2014 and intensified in the last 3 years (2020–2022). Putative virus—like disorders were inspected in 11 out of 91 samples, including leaf deformation and/or discoloration; as well as growth abnormalities and fruit deformation. Leaves from different forty-two citrus plants (Clementine, Grapefruit, Lemon, Orange, Kumquats, Pomelo, and two rootstocks, Volkameriana and Trifoliata) were collected and conserved for molecular assays.

### Molecular assays

Two-step RT-PCR was applied to detect any of citrus viroids: CEVd, CVd-IV, and CVd-III, using specific sets of primers as shown in Table 2.

**Table 2:** Sets of primers used for one step RT-PCR detection of citrus viroids.

Primer	Sequence 5→–3	Size bp	Position	Reference
CEVd-R	CCGGGGATCCCTGAAGGACTT	371	78–98	^44^
CEVd-F	GGAAACCTGGAGGAAGTCGAG		99–119	
CVd-III-R	CGTCACCAACTTAGCTGCCTTCGT	269	91–112	^45^
CVd-III-F	GTCTCCGCTAGTCGGAAAGACTCCG		135–159	
CVd-IV-R	CCGGGGATCCCTCTTCAGGT	138	52–71	^46^
CVd-IV-F	GGTGGATACAACTCTTGGGTTGT		217–239	
18s^a^-R	TTCAGCCTTGCGACCATACT	844		^47^
18s^a^-F	CGCATCATTCAAATTTCTGC			

(R) antisense primer, (F) sense primer.

^a^Internal control of plant rRNA.

Total nucleic acid (TNA) was extracted from 100 mg of citrus tissues according to Foissac et al.^48^ procedures. Briefly, leaf tissues were homogenized in grinding buffer (pH 5.6–5.8) [4M Guanidine thiosianate, 0.2 M sodium acetate, pH 5.2, 25 mM EDTA, 1.0 M potassium acetate, 2.5% PVP-40 and 2% Sodium bisulphate] with addition of 0.5% of sodium metabisulfite and 150 µl of 10% Sodium lauroyl sarcosinate (Sarkosyl) before incubating at 70°C for 10 min. On ice, 500 µl of extraction were mixed with binding solution (250 μl ethanol absolute, 500 μl NaI 6M and 35μl of re-suspended silica) and gently agitated for 10 min at room temperature. Pellets were then collected and subjected to two washing with [10.0 mM Tris–HCl, pH7.5; 0.5 mM EDTA, 50.0 mM NaCl, 50% Ethanol]. 150 μl TNA were liberated by distilled water, and NanoDrop 2000/2000c UV–Vis spectrophotometers (JENWAY, Genova Nano, Fisher Scientific UK company, England.) was used to measure their quantity and quality.

A reverse transcription reaction was carried out from 500 ng TNA and 200 units of SuperScript™ III RT (Life Technologies Corporation) in a final volume of 20 µl. Firstly, TNA were mixed with 0.5µl of random hexamers primer (50ng/µl), 0.5 µl oligodT (50 µM), and 1 µL dNTPs (10 μM) in a final volume of 10 µl. The mixture was incubated at 65 °C for 5 min. On ice, 2 µl of 10 × RT buffer, 2 µl of 0.1M DTT, 4µl of 25mM MgCl_2_, 1 µL RNaseOUT(40U/µL), and 1µL SuperScriptIII RT(200U/µL) were added to the mixture, before incubation at 25 °C/10 min followed by 50 °C/50 min. The reaction was terminated at 85 °C for 5 min and cooled at 4 °C for next use in PCR reaction.

A single PCR was performed for each viroid with its pair of primers separately (Table2). 2 μl cDNA were added to a final volume of 25 μl PCR mix composed of: 2.5μl of 10 × Taq polymerase buffer; 2 μl of 50 μM MgCl2; 0.5 μl of 10 μM dNTPs and 0.25 μl of Taq polymerase (5 unit/μl) (Promega Corporation, USA)), with primer pairs final concentrations of 0.2 μM for CVd-III and CVd-IV, and 0.5 μM for CEVd, meanwhile 18s primers were reached up to 0.14 μM. After a 5-min denaturation stage at 94 °C, 45 cycles of cDNA amplification were performed. Each cycle consisted of a 50-s denaturation stage at 94 °C followed by a 55 °C annealing step for 50 s, and 2 min extension at 72 °C. The elongation stage was at 72 °C for10 min. PCR products were visualized under UV light detector in 1.2% agarose gel stained with GelRed (Biotium) according the manufacturer’s recommended protocol.

For quick detection of several viroids with one reaction with high sensitivity, specificity and cost-effective, multiplex PCR was tested according to Wang et al.^49^. The mix of all viroid primer pairs at a final concentration of 0.2 μM except for CEVd (0.5 μM), and 18s (0.14 μM). Briefly, 2 μl cDNA mixture were used in PCR amplification with 2.5μl of 10 × Taq polymerase buffer (Promega Corporation, USA), 2 μl of 50 μM MgCl_2_, 0.5 μl of 10 μM dNTPs, and 0.25 μl of Taq polymerase (5 unit/μl) in 25 μl final volume. The cDNA amplification was done with 45 cycles after initial denaturation at 94 °C for 5 min. Each cycle consisted of: denaturation at 94 °C for 50 s, annealing at 58 °C for 50 s and extension at 72 °C for 2 min. The final extension was done at 72 °C for 10 min.

### Sequencing and data analysis

Selected isolates of the viroids (CVd-IV, CEVd, and CVd-III) (as part of a collaboration work with the National Agricultural Research Center for detecting eight of citrus viroids) were sequenced using the sequencing facilities at An-Najah National Hospital-Nablus. The sequences were searched with databases using BLASTn on the National Center for Biotechnology Information–NCBI web server. The obtained sequences were deposited at GenBank (NCBI) and their accession numbers were obtained for each. Nucleotide sequence similarity was achieved using by Pairwise Sequence Alignment-EMBOSS Water provided by European Molecular Biology Laboratory (EMBL).

### Ethics approval and consent to participate

Authors confirm that the use of plants in the present study complies with international, national and/or institutional guidelines.

## Conclusion

Three of citrus viroids (CEVd, CVd-III, and CVd-IV) had been detected in Palestinian germplasm collection, reporting their existence for the first time in the country. For quick detection of several viroids with one reaction with high sensitivity, specificity and cost-effective. Multiplex RT-PCR was recommended. Applying phytosanitary measurements including viroids was highly demanded.

### Supplementary Information

Supplementary Information 1.

Supplementary Information 2.

Supplementary Information 3.

Supplementary Information 4.

Supplementary Information 5.

## Data Availability

Adequate and clear descriptions of the applied materials and tools are provided in the materials and method section of manuscript. In addition, the obtained data is clearly justified by mentioning the figures and tables in the manuscript. The datasets generated and/or analyzed during the current study are available in the [Citrus Viroids] repository, in the link:https://drive.google.com/drive/folders/1Bv0wpKKxkRCW1C_0NXHMbiX8zZfKtBf8?usp=drive_link. The datasets used and/or analyzed during the current study available from the corresponding author on reasonable request. All data generated or analyzed during this study are included in this published article [and its Supplementary Information files].
