# Supplementary material for: Molecular detection of Citrus exocortis viroid (CEVd), Citrus viroid-III (CVd-III), and Citrus viroid-IV (CVd-IV) in Palestine
Source: Sci Rep. 2024 Jan 3;14:423. doi: 10.1038/s41598-023-50271-5 (PMC10764322; doi:10.1038/s41598-023-50271-5)
Supplement: Supplementary file 1 — Supplementary Information 1. [file 41598_2023_50271_MOESM1_ESM.pdf]

Supplementary 1. Single and mixd infection of germplasm plants

		#	***CVdIV***	***CVdIII***	***CEVd***
42	Volkameriana	8	**CVdIV**		
41	Trifoliata	10	**CVdIV**		
38	Pomelo	15	**CVdIV**		
37	Pomelo	1	**CVdIV**		**CEVd**
34	Lemon	11	**CVdIV**	**CVdIII**	**CEVd**
32	Kumquats	12	**CVdIV**		**CEVd**
30	Grapefruit	5	**CVdIV**		
28	Grapefruit	13	**CVdIV**		**CEVd**
19	Clementine	9	**CVdIV**		
18	Clementine	7	**CVdIV**		
17	Clementine	2	**CVdIV**		
16	Clementine	6	**CVdIV**		
15	Clementine	3	**CVdIV**		
12	Clementine	14	**CVdIV**	**CVdIII**	**CEVd**
1	Orange	4	**CVdIV**		**CEVd**
35	Lemon	25		**CVdIII**	**CEVd**
33	Kumquats	27			
31	Kumquats	22		**CVdIII**	**CEVd**
29	Grapefruit	19		**CVdIII**	
14	Clementine	28		**CVdIII**	
13	Clementine	18			**CEVd**
3	Orange	20		**CVdIII**	
2	Orange	23			**CEVd**
