# Supplementary material for: Molecular detection of Citrus exocortis viroid (CEVd), Citrus viroid-III (CVd-III), and Citrus viroid-IV (CVd-IV) in Palestine
Source: Sci Rep. 2024 Jan 3;14:423. doi: 10.1038/s41598-023-50271-5 (PMC10764322; doi:10.1038/s41598-023-50271-5)
Supplement: Supplementary file 2 — Supplementary Information 2. [file 41598_2023_50271_MOESM2_ESM.pdf]

Supplementary 2. Molecular detection Data

	Single	double	Multiple
Clementine	+	-	+
Grapefruit	+	+	-
Lemon	+	-	+
Orange	+	+	-
Kumquats	-	+	-
Pomelo	+	-	-
Volkameriana	+	-	-
Trifoliata	+	-	-

	No.	*CVd-IV*	*CVd-III*	*CEVd*	Single	double	Tible
Clementine	16	6	2	2	7	0	1
Orange	11	1	1	2	2	1	0
Pomelo	4	2	0	1	1	1	0
Grapefruit	3	2	1	1	2	1	0
Lemon	3	1	2	2	0	1	1
Kumquats	3	1	1	2	0	2	0
Volkameriana	1	1	0	0	1	0	0
Trifoliata	1	1	0	0	1	0	0
	42	15	7	10	14	6	2

**Citrus cultivar**		**Single**	**double**	**Multiple**
Clementine	16	+	-	+
Orange	11	+	+	-
Pomelo	4	+	-	-
Grapefruit	3	+	+	-
Lemon	3	+	-	+
Kumquats	3	-	+	-
Volkameriana	1	+	-	-
Trifoliata	1	+	-	-
