# Supplementary material for: Molecular detection of Citrus exocortis viroid (CEVd), Citrus viroid-III (CVd-III), and Citrus viroid-IV (CVd-IV) in Palestine
Source: Sci Rep. 2024 Jan 3;14:423. doi: 10.1038/s41598-023-50271-5 (PMC10764322; doi:10.1038/s41598-023-50271-5)
Supplement: Supplementary file 3 — Supplementary Information 3. [file 41598_2023_50271_MOESM3_ESM.pdf]

Supplementary file 3.Sequences

>CVdIV
CGACATCCAGAGTTGTTACCCGGAATGGCCCGCGTTTGAGACCCCTCTGG
GGAATTTCTCTGCGGGACCAAATAAAAACAGCTTGTGGAGGGAACATACC
TGAAGAGGGATCCCCGGAATCTCTCAGAAGCTTGCGATATAAATATGTTT
TACAAGCTCATCTAAAGGTTTCTCCATCTTTAGTCAAAACATTGATTTTC
TTTAAATAAGAATCTGATTCAAAATCACTACTGCTAGATGCAGAGGTATC
TGAGTCAATAAGCTCATCGACCTGAAGAGGGATCCCCGG

>CVdIII-1
TCCTCCGGCGCCCCGCTAGCTCGCCGCTAGTCGAGCGGACCACGGGAAGT
AGCCCTACTCCTAATCTGTTTTTATTTAGGCTAGAAGGGGATTGGGCCTC
CAGGGTAAAACACGATTGGTGTTTTCCCCGGAAAACTCCGTGTGGTTCCT
GTGGGGCACACCCCCTTGCCGAAAATAAAACGCAGAGAGGGAAAGGGAAC
TTACCTGTCGTCGTCGACGAAGGCAGCTAAGTTGGTGACGCCGCTAAGTT
CCCTTTCCTCTCTGCGTTTTATTTTCTGCAAGGGGGTGTGCCCCACAGGA
ACCACACGGAGTTTTCCGGGGAAAACACCAATCGTGTTTTACCCTGGAGG
CCCAATCCCTTCTTGCCTAAATAAAAACAGATTAGGAGTAGGGCTACTTC
CGTGGTCGCTGACTAGCGGCGAGCTAGCGGGTCTGCGGAGGATGCGGAGT
CTTTCCTACTAGCGGAGACAGCA

>CVdIII-2
TCCTCCGCGCCCCTCCTTGCTCGCCGCTAGTCGAGCGGACTTCAGGAGAG
TAGCCCCAATCCTAACCTGTTTTTATCTAGGCTAGAAGGGGATTGGGCCT
CCAGGGTAAAACACGATTGGTGTTTTCCCCGGGAAACTCCGTGTGGTTCC
TGTGGGGCACACCCCCTTGCCGAAAATAAAACGCAGAGAGGGAAAAGGGA
ACTTACCTGTCGTCGTCGACGAAGGCAGCTAAGTTGGTGACGACAGGTAA
GTTCCCTTTTCCCTCTCTGCGTTTTATTTTCGGCAAGGGGGTGTGCCCCA
CAGGAACCACACGGAGTTTCCCGGGGAAAACACCAATCGTGTTTTACCCT
GGAGGCCAATCCCTTCTGCCTAGATAAAAACAGGGTTAGGATTGGGGGCT
ACTCTCTGAAGTCCCTTGACTAGCGCCAAGAAGGAAGGGTCTGCCGGAAG
GATGCGGAATCTTTCCAACTAGGGGAAACAA

>CEVd
TCGCAGTCTAGTGTGTAACGGGTAAAGTCCTTCGGGATCCCCGGAGGGGA
AAACAGGAGTCGTCTCCTTCCTTTCGCTGCTGGCTCCACATCCGATCGTC
GCTGAAGCGCCACGCCCCCTCGCCCGGAGCTTCTCTCTGGCTACTACCCG
GTGGATACAACTGAAGCTTCAACCCCAAACCGCTTTTCTTATATCTTCAC
TGCTCTCCGGGCGAGGGTGAAAGCCCTCGGAACCCTAGATTGGGTCCCTC
GGGATCTTTCTTGAGGTTCCTGTGGTGCTCACCTGACCCTGCAGGCAGGA
AAAGAAAAAAGAGGCGGCGGGGAAGAAGTCCTTCAGGATCCCCGG
