# Supplementary material for: Molecular detection of Citrus exocortis viroid (CEVd), Citrus viroid-III (CVd-III), and Citrus viroid-IV (CVd-IV) in Palestine
Source: Sci Rep. 2024 Jan 3;14:423. doi: 10.1038/s41598-023-50271-5 (PMC10764322; doi:10.1038/s41598-023-50271-5)

Supplement 5

Two steps Multiplex PCR was performed to 42 RNA extract samples ,as shown in the next image

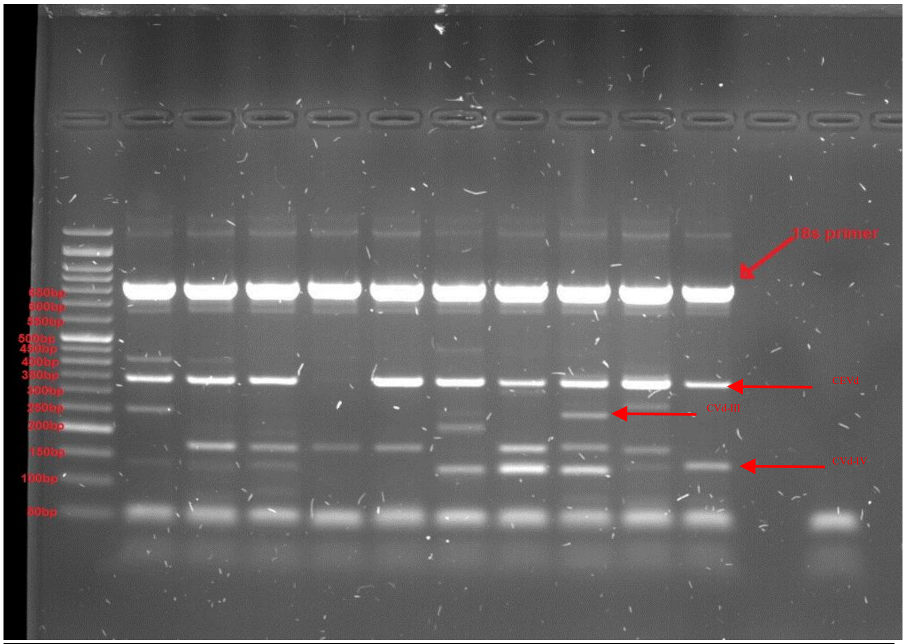

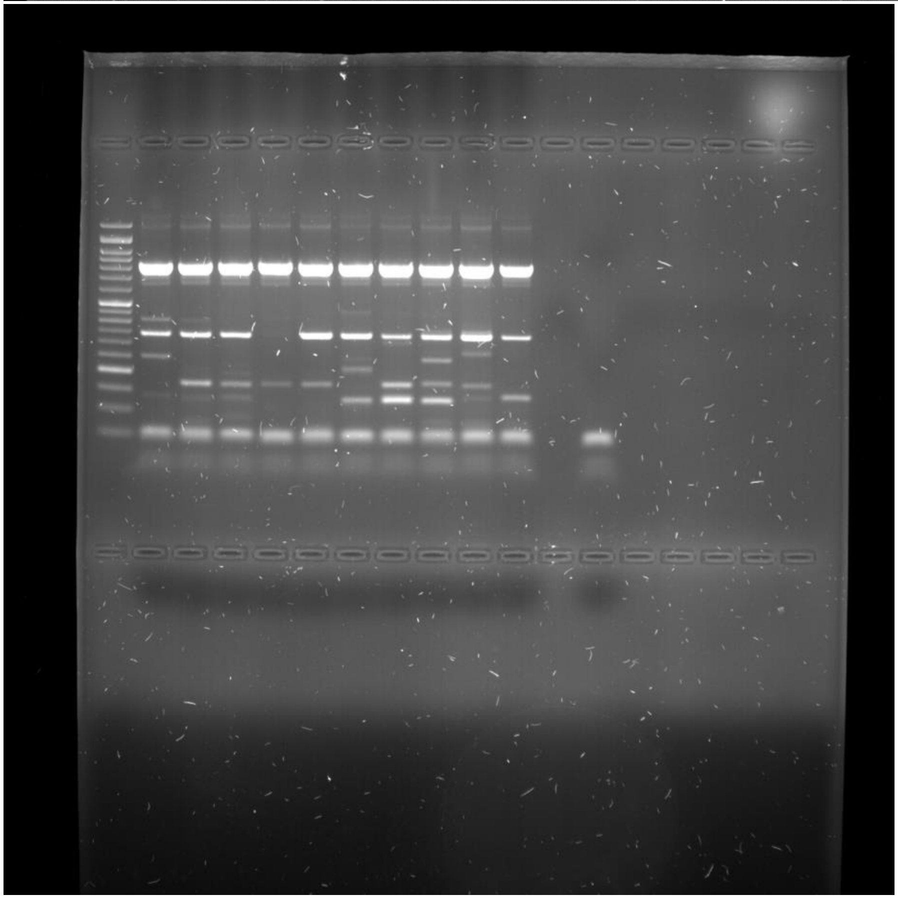

For more specifity uniplex pcr was perfomed for samples that inefecetd with singel viroid and band appeared like this

CVd IV

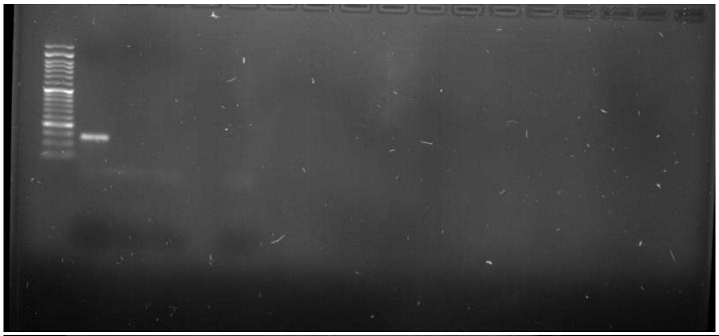

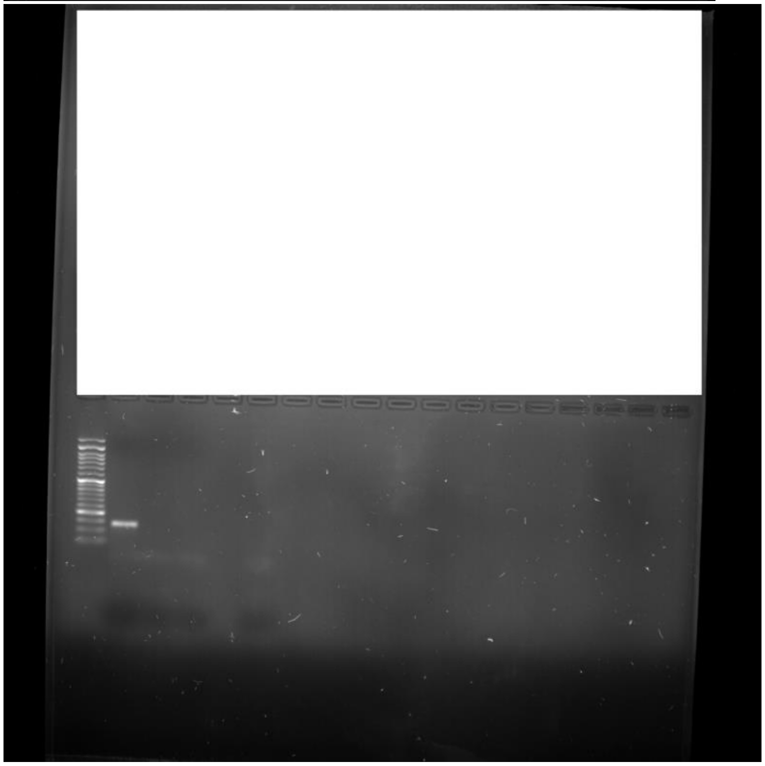

CEVd

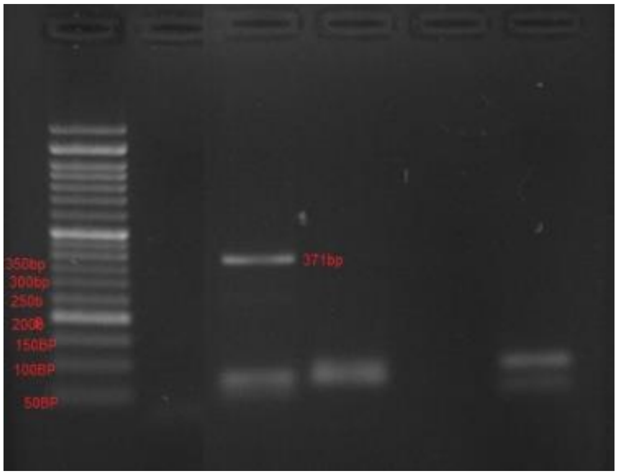

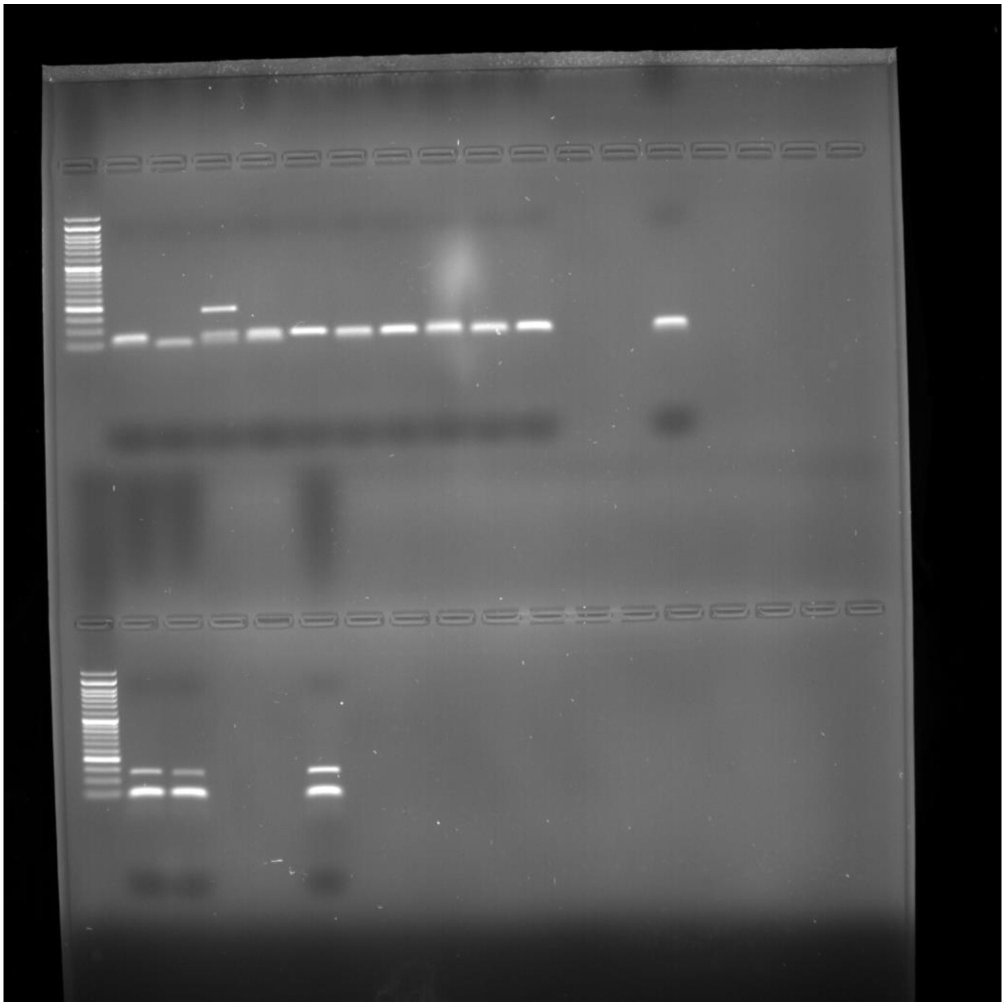

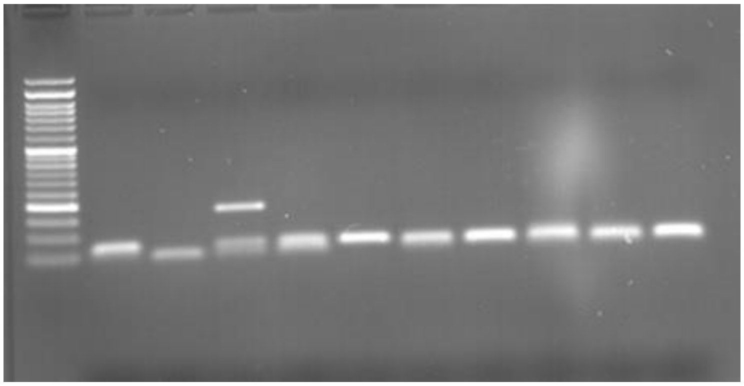

3.CVd -III

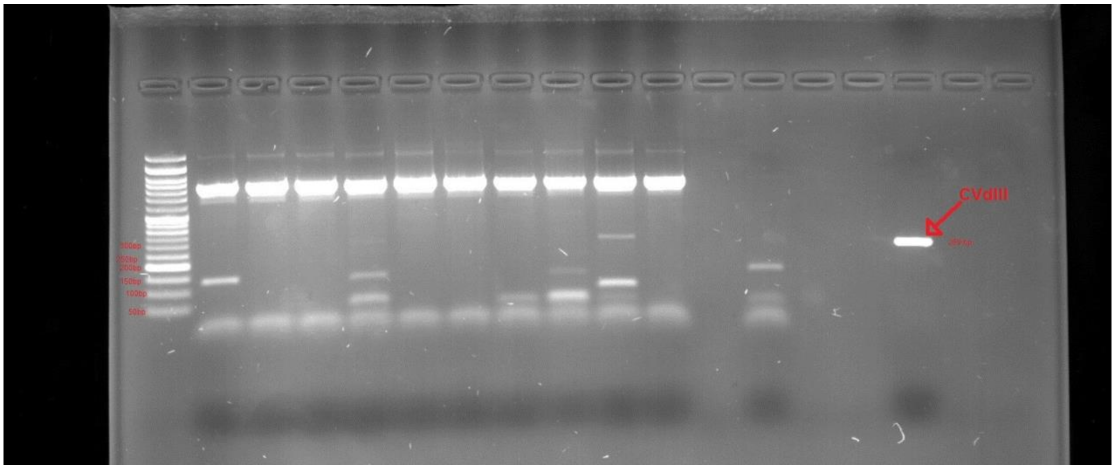

Supplement: Supplementary file 5 — Supplementary Information 5. [file 41598_2023_50271_MOESM5_ESM.pdf]
